# Comparative Study of *Nigella sativa* and Triple Therapy in Eradication of *Helicobacter pylori* in Patients with Non-Ulcer Dyspepsia

**DOI:** 10.4103/1319-3767.65201

**Published:** 2010-07

**Authors:** Eyad M. Salem, Talay Yar, Abdullah O. Bamosa, Abdulaziz Al-Quorain, Mohamed I. Yasawy, Raed M. Alsulaiman, Muhammad A. Randhawa

**Affiliations:** Department of Physiology, College of Medicine, King Faisal University, Dammam, Saudi Arabia; 1 Department of Pharmacology, College of Medicine, King Faisal University, Dammam, Saudi Arabia; 2 Department of Medicine, Division of Gastroenterology, King Fahd Hospital of the University, Al-Khobar, Saudi Arabia

**Keywords:** Amoxicillin, black seeds, clarithromycin, H. pylori, *N. sativa*, non-ulcer dyspepsia, rapid urease test, triple therapy

## Abstract

**Background/Aim:**

A large number of diseases are ascribed to *Helicobacter pylori* (*H. pylori*), particularly chronic active gastritis, peptic ulcer disease and gastric cancer. Successful treatment of *H. pylori* infection with antimicrobial agents can lead to regression of *H. pylori*–associated disorders. Antibiotic resistance against *H. pylori* is increasing, and it is necessary to find new effective agents. *Nigella sativa* seed (NS), a commonly used herb, possesses *in vitro* anti-helicobacter activity. The present study was undertaken to evaluate the efficacy of NS in eradication of *H. pylori* infection in non-ulcer dyspeptic patients.

**Materials and Methods:**

The study was conducted on 88 adult patients attending King Fahd Hospital of the University, Al-Khobar, Saudi Arabia, from 2007 to 2008, with dyspeptic symptoms and found positive for *H. pylori* infection by histopathology and urease test. Patients were randomly assigned to four groups, receiving i) triple therapy (TT) comprising of clarithromycin, amoxicillin, omeprazole [*n*= 23], ii) 1 g NS + 40 mg omeprazole (OM) [*n*= 21], iii) 2 g NS + OM [*n*= 21] or iv) 3 g NS + OM [*n*= 23]. Negative *H. pylori* stool antigen test four weeks after end of treatment was considered as eradication.

**Results:**

*H. pylori* eradication was 82.6, 47.6, 66.7 and 47.8% with TT, 1 g NS, 2 g NS and 3 g NS, respectively. Eradication rates with 2 g NS and TT were statistically not different from each other, whereas *H. pylori* eradication with other doses was significantly less than that with TT (*P* < 0.05). Dyspepsia symptoms improved in all groups to a similar extent.

**Conclusions:**

*N. sativa* seeds possess clinically useful anti-H. pylori activity, comparable to triple therapy. Further clinical studies combining *N. sativa* with antibiotics are suggested.

*Helicobacter pylori (H. pylori)* infection is extremely common worldwide, and a large number of diseases have been ascribed to *H. pylori*, particularly chronic active gastritis, peptic ulcer, gastric cancer and gastric Mucosa-Associated Lymphoid Tissue (MALT)-lymphoma.[1,2] Considering peptic ulcer alone, at least 6,000,000 new cases occur each year worldwide mainly linked to *H. pylori*. Figures regarding adenocarcinoma amount to 899,000 new cases annually, and 75% of these are probably linked to *H. pylori* infection.[3]

*H. pylori* is a gram-negative flagellated spiral bacterium which is usually acquired during childhood, and the infection persists throughout life unless specifically treated.[4] Eradication of *H. pylori* infection is recommended to prevent ulcer recurrence and complications in all patients with documented peptic ulcer disease.[5] Since *H. pylori* has a peculiar habitat and characteristics, it is difficult to eradicate eradicate with a single antibiotic[6] which is why the standard therapy includes a combination of at least two antibiotics along with a proton pump inhibitor (PPI). Nevertheless, current treatment regimens, including PPIs plus two antibiotics (usually clarithromycin and amoxicillin), fail to eradicate *H. pylori* in approximately 20% of the patients.[6] Recently, *H. pylori* has been found to be resistant to one or more of the antimicrobial drugs.[7] For example, in one of the studies the resistance was reported in 44% of cases to metronidazole and in 14% of cases to clarithromycin[8] while in another study the resistance against the same drugs was 49.4% and 10.8%, respectively.[9] In light of this emerging resistance, there is a need to look for new remedies effective against *H. pylori*. The traditional use and anecdotal evidence of plants as medicine provide the basis for suggesting that plant extracts may be useful for specific medical conditions. It has been shown that essential oils, extracted from plants, are bactericidal against *H. pylori* without the development of acquired resistance, suggesting that essential oils may have potential as new and safe agents for inclusion in anti–*H. pylori* regimens.[10]

*Nigella sativa (N. sativa)*, one of the members of Ranunculaceae family, commonly grows in the Middle East, eastern Europe and eastern and middle Asia. *N. sativa* and its oil are being used as food additives as well as natural remedies for many ailments for over thousands of years.[11] Many active ingredients have been isolated from *N. sativa*, including: thymoquinone, thymohydroquinone, dithymoquinone, thymol, carvacrol, nigellicine and alpha-hedrin.[12] In addition, many pharmacological effects of *N. sativa* and its active principles have been identified, such as immune stimulation, anti-inflammatory, anti-cancer and antimicrobial activity.[12,13]

The antibacterial effect of the phenolic fraction of *N. sativa* oil was first reported by Topozada in 1965.[14] Diethyl-ether extract of *N. sativa* has been reported to inhibit gram-positive and gram-negative bacteria, as well as pathogenic yeast.[15] Recently, crude extracts of *N. sativa* were reported to have a promising effect on multi-drug–resistant organisms, including gram-positive and gram-negative bacteria.[16] In a recent *in vitro* study, *N. sativa* extract produced within 60 minutes, a 100% inhibition of the growth of all the strains of *H. pylori* that were tested.[17]

Thus the present study aimed at the investigation of the effectiveness of *N. sativa* in eradication of *H. pylori* in non-ulcer dyspeptic patients compared to that of standard triple therapy.

## MATERIALS AND METHODS

The study was conducted in the gastroenterology endoscopy unit at King Fahd Hospital of the University (KFHU), Al-Khobar, Saudi Arabia, from March 2007 to August 2008. A total of 308 patients were initially enrolled in the study, out of which 110 were included according to the inclusion/ exclusion criteria. Of these 110 patients randomly assigned, 22 were excluded, discontinued or lost. All patients (*n* = 88; 32 male and 56 female; age range, 18-65 years) finally included in the study had complaints of dyspeptic symptoms and had positive result for *H. pylori* infection by both histopathology and rapid urease test Compylobacter-Like Organism (CLO) test. Patients were excluded if 1) the endoscopy showed peptic ulcer, gastric cancer or gastrointestinal bleeding; 2) they had taken proton-pump inhibitors, bismuth or antibiotics in the last four weeks before endoscopy; 3) they were pregnant or lactating mothers; 4) they were intolerant or allergic to therapeutic regimens; or 5) they failed to report for follow-up. The nature, aim and expected outcome of the study were explained to each patient, and written consent was obtained. The prospective study was approved by the medical and ethical committee of King Fahd Hospital of the University, Al-Khobar, Saudi Arabia, and conducted according to the guidelines of Helsinki declaration.

**Figure 1:**
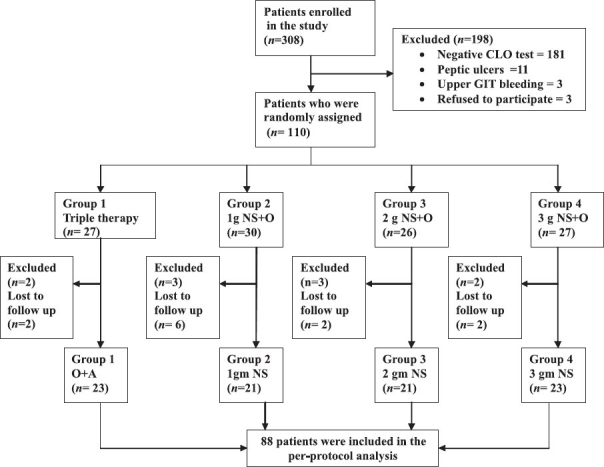
Trial flow profile

The trial profile is shown in Figure 1. The study was designed as an open, randomized clinical trial with four groups. The patients were randomly assigned to one of the four groups using alternate subject method as follows: group I: patients received triple therapy, i.e., clarithromycin tablets 500 mg twice daily and amoxicillin 1 g twice daily for one week; group II: patients received 1 g *N. sativa* powder as capsule of 500 mg (1 capsule twice daily after meals) for four weeks; group III: patients received 2 g *N. sativa* powder as capsule of 500 mg (2 capsules twice daily after meals) for four weeks; group IV: patients received 3 g *N. sativa* powder as capsule of 500 mg (2 capsules thrice daily after meals) for four weeks. Patients in all the groups received omeprazole 40 mg once daily for four weeks. Patients were instructed to avoid anti-ulcer and ulcerogenic medications and any antibiotic other than the drugs given until completion of study. They were advised to take normal balanced diet during treatment.

*N. sativa* capsules were obtained from BioExtract (Pvt.) Ltd., Sri Lanka, and each capsule contained 500 mg of ground *N. sativa L* seeds. Clarithromycin (Klacid^®^, Abott, England) 500 mg capsules, amoxicillin (Amoxil^®^, Beecham, England) 500 mg capsules and omeprazole (Gasec^®^, Mepha, Switzerland) 20 mg capsules were obtained from a local pharmacy. The doses selected for the study were based on studies being carried out in children and adults.[18,19] The doses of 1, 2 and 3 g/d were considered safe in light of the acute and chronic toxicity studies of *N. sativa* fixed oil and of thymoquinone (the active ingredient in *N. sativa*) in mice.[20,21]

Esophago-gastroduodenoscopy (EGD) was done for every patient according to the standard protocol of Gastroenterology Division at KFHU. Mucosal states of esophagus, stomach and duodenum were assessed and recorded, and mucosal biopsies were taken from the antrum and the body of the stomach. Gastritis, duodenitis were diagnosed endoscopically based on the presence of erythema, erosion and nodularity, which was later confirmed by histology. Endoscopic diagnosis of gastroesophageal reflux disease (GERD) was made according to Los Angeles (LA) classification. Mucosal biopsy specimens were placed in fixative and sent for histopathological study, while one specimen was put in rapid urease test slide (CLO test — Kimberly-Clark-Ballard Medical Products, Draper, Utah, USA) for immediate detection of *H. pylori*. The presence of *H. pylori* was defined by two positive results (histopathologic examination and rapid urease test).[22] Patients were subsequently excluded from the study if the presence of *H. pylori* determined by rapid urease test was not confirmed by histopathological findings.

A rapid monoclonal immunochromatographic method (ImmunoCard STAT HpSA, Meridian Bioscience, Europe) was used to detect the presence of *H. pylori* antigen in the stool of the patients 4 weeks after the end of treatment.[23] The test was performed according to the instructions of the manufacturers.

Eligible patients were interviewed and they underwent physical examination, including weight and height. A questionnaire regarding demographic data (name, age, sex, etc.), as well as about the presence of other illnesses or habits (smoking, alcohol, etc.), was filled. The details of relevant gastrointestinal symptoms (epigastric pain and reflux symptoms) were filled for each patient, using a special scoring system that is routinely employed in the gastroenterology unit of the hospital and is a simplified adaptation of “Short-form Leeds Dyspepsia Questionnaire”.[24]

This system evaluated three important parameters regarding every dyspeptic symptom, viz., the frequency of the that particular dyspeptic symptom (0= less than 3 times/week; 1= most days of the week), relief of the symptom (0= spontaneously relieved; 1= relieved only by medication), and effect of that particular symptom on the quality of life (0= no effect, 1= presence of effect). The effect on quality of life was assessed as to whether the symptom interferes with daily routine working of the person or not. Scores for each of the symptoms could range from zero (no symptom) to a maximum of 3 (severe symptom). In addition, the patients were followed up weekly during the four weeks of treatment with *N. sativa*, through telephone contact. The follow-up information regarding improvement of the dyspeptic symptoms, appearance of side effects and compliance of the patients was documented in the hospital file of each patient. No antibiotic or proton pump inhibitors were allowed during the two weeks preceding the stool antigen test. Finally at the end of the treatment, assessment of dyspeptic symptoms for each patient was done but endoscopy was not performed.

The primary outcome measure was eradication of *H. pylori*, which was considered to be achieved on the basis of a negative stool antigen test four weeks after the end of treatment. The secondary outcome measure included changes in the clinical condition as assessed by an improvement in dyspepsia scores.

## Statistical analysis

Only per-protocol population was included in statistical analysis. Treatment groups were compared with respect to the proportion of patients in whom eradication was successful using chi-square test (X^2^ *test*). If a statistically significant difference was detected, pair-wise comparisons were carried out using 2 × 2 chi-square contingency table with Yate’s (continuity) correction; the 95% confidence interval (95% CI) was also calculated. The different study groups were compared with respect to demographic data and endoscopic findings using ANOVA (one-way analysis of variance) for comparison of the means of age and BMI; and the chi-square (X^2^) test, for comparison of the proportions of other categorical variables (sex, smoking, endoscopic findings). The severity of dyspeptic symptoms before and after treatment was compared in each group using the Wilcoxon signed rank test. The post-treatment dyspeptic symptoms scores were compared between the four groups using Kruskal-Wallis test for ranked data. A *P* value of < 0.05 was taken as significant. All statistical analyses were performed using the Statistical Package of Social Science (SPSS) version 11.

## RESULTS

Of the 110 patients randomly assigned, 10 were excluded as they had to use antibiotics for some other ailment, and 11 discontinued because of either traveling abroad or loss of contact. One person was excluded during the course of investigation because of noncompliance. Thus 88 patients were finally recruited in the study. The treatment groups were well matched at baseline in relation to demographic and pretreatment clinical characteristics of the patients, including endoscopic findings; there were no statistically significant differences between the treatment groups [Table 1].

**Table 1:** Baseline pretreatment characteristics of the patients

Variable	Group 1 TT (*n*=23)	Group 2 1 g NS+O (*n*=21)	Group 3 2g NS+O (*n*=21)	Group 4 3 g NS+O (*n*=23)	Differences between groups (*P* value)
Demographic data					
Age: mean ± SD	41.7 ± 14.7	43.5 ± 14.6	43.5 ± 9.9	40.8 ± 11.9	0.926
BMI: mean ± SD	28.4 ± 6.2	27.9 ± 5.2	30.2 ± 7.2	28.4 ± 5.8	0.808
Sex: Males	8	8	5	11	0.592
Females	15	13	16	12	
Smokers	2	5	1	6	0.126
Endoscopic findings					
Normal endoscopy	0	0	1	1	0.290
Antral gastritis	6	10	8	8	
Pangastritis	6	4	4	5	
GERD	0	0	0	2	
Gastritis + GERD	11	5	5	6	
Gastroduodenitis + GERD	0	2	3	1	

NS: *Nigella sativa*; O: Omeprazole; GERD: gastroesophageal refl ux disease (according to LA classification), n: number of patients; SD: standard deviation. Oneway analysis of variance (ANOVA) for comparison of the means of age and BMI. Chi-square (χ^2^) test for comparison of the proportions of categorical variables (sex, smoking, endoscopic findings). There were no signifi cant differences between the four groups regarding age, sex, BMI, smoking and endoscopic findings

None of the patients withdrew from the study because of side effects or otherwise. Compliance was similar in all groups, and more than 98% of the patients took all the medications correctly. Only 1 patient was erratic in taking the medications, as assessed by frequent telephonic contact and counting the number of capsules remaining at the last visit.

Table 2 shows the eradication rates of different therapies in various groups of patients. The eradication of H. pylori was significantly better with TT compared to 1 g/d and 3 g/d of *N. sativa*, while it was statistically comparable to 2 g/d of *N. sativa* used for four weeks along with omeprazole.

**Table 2:** Comparison of *H. pylori* stool antigen test (HpSA) results four weeks after the end of treatments with triple therapy and different doses of *Nigella sativa*

Group	*H. pylori* stool antigen test results		Eradication	Difference from triple therapy
	Patients with negative HpSA	Patients with positive HpSA	Rate %	*P* value (95% CI)
Triple therapy				
(A+C +O) (n=23)	19	4	82.6	‐
1g NS+ O (*n*=21)	10	11	47.6*	0.033 (0.068 ‐ 0.569)
2g NS+O (*n*=21)	14	7	66.7	0.384 (‐0.096 ‐ 0.396)
3g NS+O (*n*=23)	11	12	47.8*	0.030 (0.072 - 0.561)

O: Omeprazole, A: Amoxicillin, C: Clarithromycin, NS: Nigella sativa. n= number of patients; CI= confidence interval.

* signifi cant differences compared to triple therapy (*P* < 0.05) using 2 × 2 chi-square contingency table with Yate’s (continuity) correction

As shown in Tables 3 and 4, the treatment regimens led to significant improvement of dyspeptic symptoms in 65%, 81%, 62% and 65% of patients in groups taking TT, 1 g/d, 2 g/d and 3 g/d of *N. sativa*, respectively (*P* < 0.001). None of the patients reported any worsening of the symptoms in any group. However, the comparison of post-treatment dyspeptic symptoms between the four groups did not reveal any significant difference.

**Table 3:** Patients showing improvement in post-treatment score of epigastric pain with respect to pretreatment score in each study group

Groups	Number of patients showing change in epigastric pain score			*P* value
	Improved score	Same score	Worsened score	
Triple therapy (*n*=23)	15 (65.2)	8	0	0.000*
1g NS+O (*n*=21)	17 (80.9)	4	0	0.000*
2g NS+O (*n*=21)	13 (61.9)	8	0	0.001*
3g NS+O (*n*=23)	15 (65.2)	8	0	0.000*

NS: *N. sativa*; O: Omeprazole; Triple therapy= (Amoxicillin + Clarithromycin + Omeprazole). n: number of patients.

* All groups showed signifi cant improvement in post-treatment score compared to the pretreatment score (*P* < 0.05) using Wilcoxon rank test

**Table 4:** Patients showing improvement in post-treatment score of reflux symptoms with respect to pretreatment score in each study group

Groups	Number of patients showing change in refl ux symptoms score			*P* value
	Improved score *n* (%)	Same score *n*	Worsened score *n*	
Triple therapy (*n*=23)	15 (65.2)	8	0	0.000*
1g NS+O (*n*=21)	12 (57.1)	9	0	0.001*
2g NS+O (*n*=21)	17 (80.9)	4	0	0.000*
3g NS+O (n=23)	17 (73.9)	6	0	0.000*

NS: *N. sativa*; O: Omeprazole; Triple therapy= (Amoxicillin + Clarithromycin + Omeprazole). n: number of patients. All groups showed signifi cant improvement in post-treatment score compared to the pretreatment score (*P* < 0.05) using Wilcoxon rank test

Adverse effects recorded in the patients taking *N. sativa* and antibiotics were minor, similar, mostly related to gastrointestinal irritation and did not persist for long.

## DISCUSSION

The rising prevalence of *H. pylori* antibiotic resistance emphasizes the need for discovery of new and safe modalities for treatment of *H. pylori* infection and the resulting gastroduodenal disease. Although *H. pylori* is susceptible to many antibiotics and plant products in vitro, not all can be used *in vivo* to eradicate the infection because of the highly peculiar characteristics and habitat of the organism and low pH of the stomach.[6]

The present study was aimed at investigating the effect of *N. sativa*, supplied as ground seeds, in three doses (1 g/d, 2 g/d and 3 g/d, for 4 weeks) on *H. pylori* eradication in humans. The results showed that a dose of 2 g/d eradicated *H. pylori* in infected patients in a proportion (67%) which is close to that of standard triple therapy. However, 1 g/d and 3 g/d *N. sativa* were significantly less effective than the triple therapy (*P* < 0.05) [Table 2] but still effective in approximately 48% of the patients. The triple therapy group in the present study achieved the highest rate for eradication of *H. pylori* (82.6%), which lies in the range reported by many recent studies investigating the efficacy of different triple therapy combinations.[5] The most effective and well-tolerated combination consists of amoxicillin, clarithromycin and omeprazole. Investigators have reported an eradication rate ranging from below 80% up to approximately 90%.[25,26] In general practice, 20% to 30% of therapies fail probably due to resistance or noncompliance of patients. These differences among various countries, despite using the same regimen, may be attributed to the presence or absence of pretreatment antimicrobial resistance.[27]

Cure of *H. pylori* is not easy, as the bacteria are located below the mucus layer, adherent to gastric mucosa where the access of antimicrobial drugs is limited; this requires a combination of antibiotics, often with additional non-antibiotic adjunctive agents. Cure rates with single antimicrobial agent (monotherapy) or single antimicrobial plus adjunctive agent such as proton pump inhibitor (dual therapy) are generally unsatisfactory. Eradication rates ranging between 53% and 57% have been reported with dual therapy employing a single antimicrobial agent plus proton pump inhibitor.[28] An improvement in the eradication rates occurred when two antimicrobial agents were used together with proton pump inhibitor (triple therapy). Comparing the eradication rates achieved by even 1 g/d *N. sativa* combined with omeprazole 40 mg daily (47.6%) and 3 g/d *N. sativa* with omeprazole 40 mg daily (47.8%), the results are similar to the eradication rate achieved by dual therapies reported in those studies. Furthermore, single or dual therapy is associated with rapid development of antibiotic resistance.[28,29] The extract of Chinese medicinal plant Goshuyu-to has been shown to enhance the effect of amoxicillin combined with omeprazole by 20%; omeprazole and amoxicillin alone resulted in 60% eradication of *H. pylori*, while addition of the extract of Goshuyu-to increased the eradication rate to 80%[30]

Two patients recruited in our study were still positive for *H. pylori* after two consecutive triple therapy courses. These patients changed to negative after receiving *N. sativa* treatment in a dose of 3 g/d along with 40 mg omeprazole for four weeks. Though a separate study is required to look into the details, this observation, together with the reported antibacterial effect of *N. sativa* against many drug-resistant bacteria (e.g., Cholera, *E. coli*, Shigella)[16] opens up the possibility of using *N. sativa* against drug-resistant *H. pylori*. *N. sativa* has also been shown to produce synergistic and additive effect with several antibiotics in vitro.[15] Thus combination of *N. sativa* with antibiotics could decrease the possibility of emergence of resistant colonies of *H. pylori* and improve the efficacy of antibiotic regimen.

The results reported here indicate a strong effect (67%) of 2 g/d *N. sativa* on the eradication of *H. pylori*. This is the first clinical trial reporting such an effect in humans. An in vitro study previously showed a 100% inhibition of growth of *H. pylori* in culture.[17] Many in vitro studies have tested the use of plants for treating *H. pylori* in an attempt to overcome the rising antibiotic resistance, and a number of them have shown anti-*Helicobacter pylori* activity.[31,32] The encouraging results of these in vitro studies await clinical trials before they can be put into use for eradicative treatment of *H. pylori* infection. *In vivo* studies exploring natural remedies for eradication of *H. pylori* are limited. Broccoli sprouts consumed twice daily for seven days have been shown to be associated with eradication of *H. pylori* in 6 (66.7%) out of 9 patients tested.[33]

Surprisingly, the *H. pylori* eradication rate with *N. sativa* 3 g/d (47.8%) was lesser than that with *N. sativa* 2 g/d (66.7%). Similar results have been reported in an *in vitro* study of *N. sativa* extract, where lower concentration exerted more antibacterial action as compared to higher concentration.[16] Moreover, doubling the dosage of clarithromycin has been reported to decrease the eradication rates *of H. pylori* from 70% to 62%.[34] One possible explanation for this observation could be that the whole ground seeds of *N. sativa* contain numerous ingredients which could have counteracted the anti-*H. pylori* effect when given in higher doses. Another possible explanation is based on the hypothesis that profound acid suppression affects the pattern and distribution of gastritis; the decrease in acid production causes shifting of *H. pylori* from the antrum to involve the whole body of stomach.[35] Since *N. sativa* exhibits a potent anti-secretory effect,[36] the sum of the actions of high dose *N. sativa* and omeprazole could have led to profound acid secretion suppression. This would enhance the spread of the bacteria to the body, causing drop in the eradication rate.

The mechanism of action of *N. sativa* is not clear. *N. sativa* seeds contain a number of essential oils, including thymoquinone, dihydrothymoquinone and terpenes. Essential oils obtained from many herbs are considered to possess antimicrobial activity, which is ascribed to their ability to disrupt the lipid structure of cell membrane.[37]

The dyspeptic symptoms improved in all the three groups of *N. sativa* to the same extent as in triple therapy; there were no significant differences between the four groups of the study in improving the dyspeptic symptoms score [Tables 3 and 4]. Reduction in dyspeptic symptoms was more likely due to the action of omeprazole rather than the effect of *N. sativa*, because all groups included omeprazole in the same dose (40 mg/day). Omeprazole and other PPIs are documented to reduce the symptoms in functional dyspepsia as a result of decreasing the gastric acid secretion and increasing the stomach pH.[38,39] Though our study could not link *N. sativa* with improvement of dyspepsia symptoms, there are recent studies which show that aqueous suspension of N. sativa possessed anti-secretory effects and exhibited a protective role for gastric mucosa against injury induced by necrotizing agents.[36] *N. sativa* oil and thymoquinone were also found to have protective effects on gastric mucosa in rats against acute alcohol-induced injury, as well as injury induced by ischemia/ reperfusion.[40,41] One probable explanation for not observing any beneficial effect of *N. sativa* on dyspepsia symptoms in our study could be the relatively smaller number of patients included in the study. Another probability is that the instrument used for assessment of symptoms was very simplified and thus could not bring out the differences clearly. Nevertheless, further human studies are suggested to figure out the beneficial effects of *N. sativa*, if present, on the dyspeptic symptoms.

## CONCLUSIONS

*N. sativa* ground seeds in a dose of 2 g/d when given along with 40 mg/d omeprazole possess clinically useful anti-*Helicobacter pylori* activity, comparable to that of the standard triple therapy. The doses of 1 g/d and 3 g/d of *N. sativa* were less effective, but the *H. pylori* eradication rate achieved with these doses was similar to that obtained with a single antibiotic. Further clinical trials employing *N. sativa* in combination with one of the antibiotics commonly used for eradication of *H. pylori* could prove to provide more efficient therapy that is relatively safer and less expensive. The improvement of dyspeptic symptoms in our study was most probably due to the proton pump inhibitor, omeprazole, though *N. sativa* has previously been shown to possess gastroprotective and anti-secretory activities.

## References

[1] Hocker M, Hohenberger P (2003). Helicobacter pylori virulence factors--one part of a big picture. Lancet.

[2] Kusters JG, van Vliet AH, Kuipers EJ (2006). Pathogenesis of *Helicobacter pylori* infection. Clin Microbiol Rev.

[3] Parsonnet J (1998). *Helicobacter pylori*: the size of the problem. Gut.

[4] Frenck RW, Clemens J (2003). Helicobacter in the developing world. Microbes Infect.

[5] Malfertheiner P, Megraud F, O’Morain C, Bazzoli F, El-Omar E, Graham D (2007). Current concepts in the management of *Helicobacter pylori* infection: the Maastricht III Consensus Report. Gut.

[6] Gerrits MM, van Vliet AH, Kuipers EJ, Kusters JG (2006). *Helicobacter pylori* and antimicrobial resistance: molecular mechanisms and clinical implications. Lancet Infect Dis.

[7] Duck WM, Sobel J, Pruckler JM, Song Q, Swerdlow D, Friedman C, Sulka A (2004). Antimicrobial resistance incidence and risk factors among *Helicobacter pylori*-infected persons, United States. Emerg Infect Dis.

[8] Savarino V, Zentilin P, Pivari M, Bisso G, Raffaella Mele M, Bilardi C (2000). The impact of antibiotic resistance on the efficacy of three 7-day regimens against *Helicobacter pylori*. Aliment Pharmacol Ther.

[9] Wang WH, Wong BC, Mukhopadhyay AK, Berg DE, Cho CH, Lai KC (2000). High prevalence of *Helicobacter pylori* infection with dual resistance to metronidazole and clarithromycin in Hong Kong. Aliment Pharmacol Ther.

[10] Ohno T, Kita M, Yamaoka Y, Imamura S, Yamamoto T, Mitsufuji S (2003). Antimicrobial activity of essential oils against *Helicobacter pylori*. Helicobacter.

[11] Salem ML (2005). Immunomodulatory and therapeutic properties of the *Nigella sativa* L. seed. Int Immunopharmacol.

[12] Khan MA (1999). Chemical composition and medicinal properties of *Nigella sativa* Linn. Inflammopharmacology.

[13] Ali BH, Blunden G (2003). Pharmacological and toxicological properties of *Nigella sativa*. Phytother Res.

[14] Toppozada HH, Mazloum HA, el-Dakhakhny M (1965). The antibacterial properties of the *Nigella sativa* l. seeds. Active principle with some clinical applications. J Egypt Med Assoc.

[15] Hanafy MS, Hatem ME (1991). Studies on the antimicrobial activity of *Nigella sativa* seed (black cumin). J Ethnopharmacol.

[16] Morsi NM (2000). Antimicrobial effect of crude extracts of *Nigella sativa* on multiple antibiotics-resistant bacteria. Acta Microbiol Pol.

[17] O’Mahony R, Al-Khtheeri H, Weerasekera D, Fernando N, Vaira D, Holton J (2005). Bactericidal and anti-adhesive properties of culinary and medicinal plants against *Helicobacter pylori*. World J Gastroenterol.

[18] Akhtar MS, Riffat S (1991). Field trial of Saussurea lappa roots against nematodes and *Nigella sativa* seeds against cestodes in children. J Pak Med Assoc.

[19] Bamosa AO, Ali BA, Sowayan SA (1997). Effect of oral ingestion of *Nigella sativa* seeds on some blood parameters. Saudi Pharm J.

[20] Zaoui A, Cherrah Y, Mahassini N, Alaoui K, Amarouch H, Hassar M (2002). Acute and chronic toxicity of *Nigella sativa* fixed oil. Phytomedicine.

[21] Badary OA, Al-Shabanah OA, Nagi MN, Al-Bekairi AM, Almazar MM (1998). Acute and subchronic toxicity of thymoquinone in mice. Drud Develop Res.

[22] Hirschl AM, Makristathis A (2007). Methods to detect *Helicobacter pylori*: from culture to molecular biology. Helicobacter.

[23] Gisbert JP, de la Morena F, Abraira V (2006). Accuracy of monoclonal stool antigen test for the diagnosis of H. pylori infection: a systematic review and meta-analysis. Am J Gastroenterol.

[24] Fraser A, Delaney BC, Ford AC, Qume M, Moayyedi P (2007). The Short-Form Leeds Dyspepsia Questionnaire validation study. Aliment Pharmacol Ther.

[25] Laheij RJ, Rossum LG, Jansen JB, Straatman H, Verbeek AL (1999). Evaluation of treatment regimens to cure *Helicobacter pylori* infection--a meta-analysis. Aliment Pharmacol Ther.

[26] Ulmer HJ, Beckerling A, Gatz G (2003). Recent use of proton pump inhibitor-based triple therapies for the eradication of H pylori: a broad data review. Helicobacter.

[27] Fischbach LA, Goodman KJ, Feldman M, Aragaki C (2002). Sources of variation of *Helicobacter pylori* treatment success in adults worldwide: a meta-analysis. Int J Epidemiol.

[28] Schwartz H, Krause R, Sahba B, Haber M, Weissfeld A, Rose P (1998). Triple versus dual therapy for eradicating *Helicobacter pylori* and preventing ulcer recurrence: a randomized, double-blind, multicenter study of lansoprazole, clarithromycin, and/or amoxicillin in different dosing regimens. Am J Gastroenterol.

[29] Peterson WL, Graham DY, Marshall B, Blaser MJ, Genta RM, Klein PD (1993). Clarithromycin as monotherapy for eradication of *Helicobacter pylori*: a randomized, double-blind trial. Am J Gastroenterol.

[30] Higuchi K, Arakawa T, Ando K, Fujiwara Y, Uchida T, Kuroki T (1999). Eradication of *Helicobacter pylori* with a Chinese herbal medicine without emergence of resistant colonies. Am J Gastroenterol.

[31] Ndip RN, Malange Tarkang AE, Mbullah SM, Luma HN, Malongue A, Ndip LM (2007). *In vitro* anti-*Helicobacter pylori* activity of extracts of selected medicinal plants from North West Cameroon. J Ethnopharmacol.

[32] Zaidi SF, Yamada K, Kadowaki M, Usmanghani K, Sugiyama T (2009). Bactericidal activity of medicinal plants, employed for the treatment of gastrointestinal ailments, against *Helicobacter pylori*. J Ethnopharmacol.

[33] Galan MV, Kishan AA, Silverman AL (2004). Oral broccoli sprouts for the treatment of *Helicobacter pylori* infection: a preliminary report. Dig Dis Sci.

[34] Bigard MA, Delchier JC, Riachi G, Thibault P, Barthelemy P (1998). One-week triple therapy using omeprazole, amoxycillin and clarithromycin for the eradication of *Helicobacter pylori* in patients with non-ulcer dyspepsia: influence of dosage of omeprazole and clarithromycin. Aliment Pharmacol Ther.

[35] Schenk BE, Kuipers EJ, Nelis GF, Bloemena E, Thijs JC, Snel P (2000). Effect of Helicobacter pylori eradication on chronic gastritis during omeprazole therapy. Gut.

[36] Al Mofleh IA, Alhaider AA, Mossa JS, Al-Sohaibani MO, Al-Yahya MA, Rafatullah S (2008). Gastroprotective effect of an aqueous suspension of black cumin *Nigella sativa* on necrotizing agents-induced gastric injury in experimental animals. Saudi J Gastroenterol.

[37] Sikkema J, de Bont JA, Poolman B (1995). Mechanisms of membrane toxicity of hydrocarbons. Microbiol Rev.

[38] Bolling-Sternevald E, Lauritsen K, Aalykke C, Havelund T, Knudsen T, Unge P (2002). Effect of profound acid suppression in functional dyspepsia: a double-blind, randomized, placebo-controlled trial. Scand J Gastroenterol.

[39] Talley NJ, Lauritsen K (2002). The potential role of acid suppression in functional dyspepsia: the BOND, OPERA, PILOT, and ENCORE studies. Gut.

[40] Kanter M, Demir H, Karakaya C, Ozbek H (2005). Gastroprotective activity of *Nigella sativa* L oil and its constituent, thymoquinone against acute alcohol-induced gastric mucosal injury in rats. World J Gastroenterol.

[41] El-Abhar HS, Abdallah DM, Saleh S (2003). Gastroprotective activity of *Nigella sativa* oil and its constituent, thymoquinone, against gastric mucosal injury induced by ischaemia/reperfusion in rats. J Ethnopharmacol.

